# Molecular Composition of Genomic *TMPRSS2-ERG* Rearrangements in Prostate Cancer

**DOI:** 10.1155/2019/5085373

**Published:** 2019-12-12

**Authors:** Manuela Krumbholz, Abbas Agaimy, Robert Stoehr, Maximilian Burger, Sven Wach, Helge Taubert, Bernd Wullich, Arndt Hartmann, Markus Metzler

**Affiliations:** ^1^Department of Pediatrics, University Hospital Erlangen, 91054 Erlangen, Germany; ^2^Department of Pathology, University Hospital Erlangen, 91054 Erlangen, Germany; ^3^Department of Urology, University of Regensburg, Caritas St. Josef Medical Center, 93053 Regensburg, Germany; ^4^Department of Urology and Pediatric Urology, University Hospital Erlangen, 91054 Erlangen, Germany

## Abstract

There is increasing interest in the use of cell-free circulating tumor DNA (ctDNA) as a serum marker for therapy assessment in prostate cancer patients. Prostate cancer is characterized by relatively low numbers of mutations, and, in contrast to many other common epithelial cancers, commercially available single nucleotide mutation assays for quantification of ctDNA are insufficient for therapy assessment in this disease. However, prostate cancer shares some similarity with translocation-affected mesenchymal tumors (e.g., leukemia and Ewing sarcoma), which are common in pediatric oncology, where chromosomal translocations are used as biomarkers for quantification of the tumor burden. Approximately 50% of prostate cancers carry a chromosomal translocation resulting in generation of the *TMPRSS2-ERG* fusion gene, which is unique to the tumor cells of each individual patient because of variability in the fusion breakpoint sites. In the present study, we examined the structural preconditions for *TMPRSS2-ERG* fusion sites in comparison with mesenchymal tumors in pediatric patients to determine whether the sequence composition is suitable for the establishment of tumor-specific quantification assays in prostate cancer patients. Genomic repeat elements represent potential obstacles to establishment of quantification assays, and we found similar proportions of repeat elements at fusion sites in prostate cancer to those reported for mesenchymal tumors, where genomic fusion sequences are established as biomarkers. Our data support the development of the *TMPRSS2-ERG* fusion gene as a noninvasive tumor marker for therapy assessment, risk stratification, and relapse detection to improve personalized therapy strategies for patients with prostate cancer.

## 1. Introduction

Prostate cancer is a common tumor in men and a highly heterogeneous disease that can vary from low-risk lesions to highly aggressive tumors [1–3]. Prostate cancer also exhibits substantial heterogeneity at the genetic level, which is reflected in chromosomal rearrangements, copy number gains or losses, and somatic mutations. Somatic mutations are preferentially detected in advanced or metastatic tumors; hence, they are unsuitable for monitoring primary, nonmetastatic disease [4, 5], whereas chromosomal rearrangements represent an early event in pathogenesis [6–8]. The most common chromosomal rearrangement in prostate cancer results in the fusion of the androgen-regulated gene, *TMPRSS2* (chr21q22.2), with ETS-related gene, *ERG* (chr21q22.3), which is present in approximately 50% of patients [9]; due to the high incidence of prostate cancer, this is the most prevalent fusion gene in human cancer [10].

Therapy monitoring and tumor surveillance control in prostate cancer are mainly based on quantification of serum levels of prostate-specific antigen (PSA); however, due to a high rate of false positive results, the benefits of the PSA as a serum marker are the subject of controversial discussion [11]. Overtreatment is a clinical challenge in local prostate cancer and exposes patients to unnecessary morbidity.

A number of novel noninvasive biomarkers, isolated from blood or urine samples, are currently under investigation for use in personalized risk stratification of patients with prostate cancer. Such markers include adipocytokines like omentin [12], fatty acid binding protein 5 (FABP5), and granulin [13]; miRNAs [14]; circulating tumor cells (CTCs) [15–17]; and plasma or urine-derived cell-free RNA and DNA. Although total nucleic acid concentrations in plasma and urine samples are not reliable biomarkers for assessment of tumor burden [18, 19], the quantification of tumor-specific mutations or copy number variations appears to be more promising [20–24].

The establishment of fusion gene assays for therapy assessment in cancer patients is more complex compared with the development of single nucleotide mutation detection assays, which are commercially available for a large number of recurrent mutations. However, fusion genes may be an advantageous target for those tests due to the high clonal stability of genomic fusion sequences. The quantification of fusion genes at the level of circulating tumor DNA (ctDNA) is superior to RNA-based assays because of the higher stability of cell-free DNA in blood plasma and urine samples. We previously demonstrated the benefit of DNA-based therapy assessment using patient-specific genomic fusion sequences for mesenchymal tumors including leukemia, lymphoma, and Ewing sarcoma (EWS) [25–28].

Here, we evaluate the *TMPRSS2-ERG* fusion gene in prostate cancer tissue, as representative of a translocation-positive epithelial tumor, for a putative application as a noninvasive ctDNA biomarker. To this end, we studied the structure and distribution of genomic *TMPRSS2-ERG* breakpoints and compared the results with data derived from mesenchymal malignancies.

## 2. Materials and Methods

### 2.1. Patients and Material

The tumor material included in our study was derived from 24 patients with *ERG* rearrangement-positive prostate cancer. These patients were identified using immunohistochemistry for detection of nuclear *ERG* overexpression. The median age at diagnosis was 67.5 years (range, 56–74 years). Written informed consent was obtained from all patients, in accordance with the Declaration of Helsinki. The study is based on the approvals of the Ethics Committees of the University Hospital Regensburg (No. 05/16) and the University Hospital Erlangen (No. 3755, dated Feb. 2008).

DNA was extracted from fresh frozen tumor tissue using the QIAamp DNA Blood Mini Kit (Qiagen), according to the protocol for DNA purification from tissue samples. To demonstrate sufficient DNA quality for long-range PCR, tumor DNA was tested by amplification of an 11.4 kb region of the single copy gene, *BCR*, on chromosome 22.

### 2.2. Identification of Patient-Specific Genomic TMPRSS2-ERG Fusion Sequences

**Figure 1:**
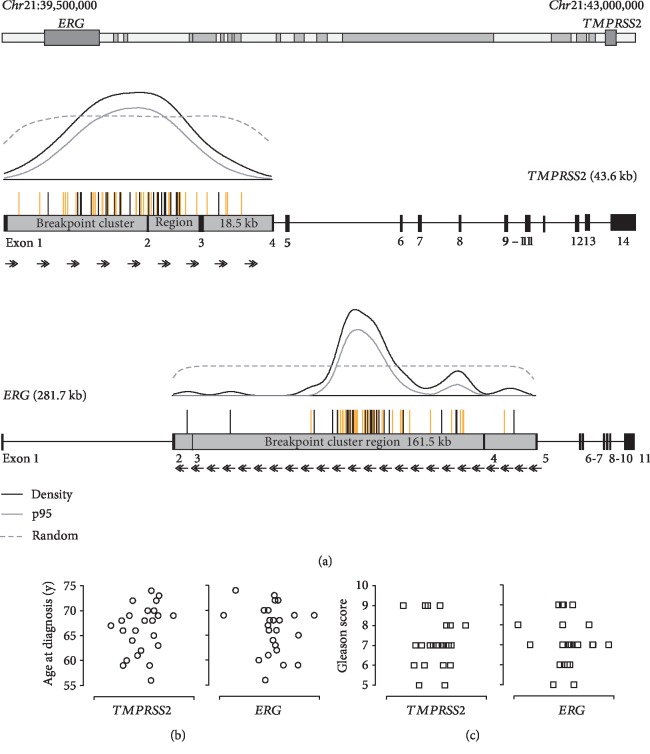
(a) Upper panel: sectional map of chromosome 21:39,500,000–43,000,000 showing all genes (gray boxes), including *ERG* and *TMPRSS2*. Middle and lower panels: genomic organization of the *TMPRSS2* and *ERG* genes and their corresponding breakpoint cluster regions, respectively. Vertical bars above the breakpoint cluster regions represent individual genomic breakpoints for the 58 patients with prostate cancer: black line, fusion sequence identified in the present study; orange line, fusion sequence reported in the literature [29–32]. Results of Kernel density analysis are illustrated above the middle and lower panels: black line, breakpoint density; gray line, lower limit of 95% confidence interval, determined using a bootstrapping procedure; dashed line, 95% confidence interval of a density function resulting from simulations at randomly distributed pseudo-breakpoints. (b) Correlation between breakpoint localization within breakpoint cluster regions of *TMPRSS2* and *ERG*, respectively, and age at diagnosis. (c) Correlation between breakpoint localization within breakpoint cluster regions of *TMPRSS2* and *ERG*, respectively, and Gleason score.

Genomic *TMPRSS2-ERG* fusion sequences were amplified using two rounds of multiplex long-range PCR (MLR-PCR), with nine forward nested primer pairs covering the breakpoint cluster region of the *TMPRSS2* gene (18.5 kb), and 24 reverse nested primer pairs covering the breakpoint cluster region of the *ERG* gene (161.5 kb) (Figure 1). Primer sequences are shown in Supplemental [Supplementary-material supplementary-material-1].

To minimize nonspecific amplification products, both *TMPRSS2* and *ERG* primers were separated into two sets each. Accordingly, we started with four different first round MLR-PCR reactions: *TMPRSS2* primer sets 1 and 2 were each combined with *ERG* primer set 1 or 2. For first round PCR reactions, external primers were used. PCR conditions were optimized using DNA from the *TMPRSS2-ERG*-positive prostate cancer cell line, VCaP. All MLR-PCR assays were performed using the AccuPrime™ Taq DNA Polymerase System (Thermo Fisher Scientific), according to the manufacturer's instructions, with 100 ng template DNA. If no specific amplicon was visible after the first round of PCR, 1 *μ*l of each reaction product was transferred as template to a second round MLR-PCR, with the corresponding nested (internal) primers.

MLR-PCR sets that generated specific amplification products were examined in more detail. To identify the *ERG* primer positioned next to the fusion site, and therefore responsible for the amplification product, a series of single PCRs with separate *ERG* primers were prepared. Aliquots (1 *μ*l) of reaction products from the first round PCR were combined with all internal nested *TMPRSS2* primers from the MLR-PCR primer set, and one of each corresponding internal *ERG* primers. The *ERG* primer that generated a specific amplification product was used in a further series of single PCRs, in combination with each of the *TMPRSS2* internal primers, to identify the *TMPRSS2* primer located closest to the fusion site. Amplified products were confirmed by an independent PCR using the identified specific primer sets adjacent to the patient's fusion site and 50 ng tumor DNA. Subsequently, PCR products were purified using the QIAquick PCR Purification Kit (Qiagen) and sequenced (Eurofins Genomics).

### 2.3. Analysis of Breakpoint Distribution and Breakpoint Characteristics

Genomic *TMPRSS2-ERG* fusion sequences were aligned to the human genome (GRCh37/hg19) using the nucleotide BLAST tool (https://blast.ncbi.nlm.nih.gov/Blast.cgi) to determine patient-specific DNA breakpoints (Table 1). For comparison and detailed characterization of genomic *TMPRSS2-ERG* fusion sites, we analyzed fusion genes in our cohort, and compared and combined the data with that from previously published fusion sequences (Weier et al., *n* = 26; Haffner et al., *n* = 3; Demichelis et al., *n* = 1; and Liu et al., *n* = 4) [29–32]. Kernel density analysis was performed using components of the free software environment R (https://www.r-project.org) to determine genomic breakpoint distribution, as described previously [33]. Repeat elements were identified using the RepeatMasker tool (http://www.repeatmasker.org). More than 20 genomic sequence motifs described as showing low DNA stability, or that were associated with DNA cleavage or rearrangement, were identified using VectorNTI software (Supplemental [Supplementary-material supplementary-material-1]). Palindromic regions were detected using the EMBOSS explorer tool (http://emboss.bioinformatics.nl). Fisher's exact test was used to determine whether there was significant colocalization of patient-specific fusion sites and repeat elements, or recombination-related DNA sequence motifs.

## 3. Results

### 3.1. Distribution of Genomic TMPRSS2 and ERG Breakpoints

Genomic *TMPRSS2-ERG* breakpoints in the prostate cancer cell line, VCap, and 24 prostate cancer patients are listed in Table 1. Kernel density analyses of a total of 58 genomic *TMPRSS2-ERG* fusion sites, including data generated in this study and previously published results [29–32], revealed a uniform distribution of genomic breakpoints within the *TMPRSS2* breakpoint cluster region (18.5 kb) with a significant accumulation of breakpoints within a 4.4 kb region (chr21:42,873,136–42,868,746) flanking exon 2 (Figure 1(a)). Breakpoints in the large *ERG* breakpoint cluster region (161.5 kb) showed significant clustering within a 21.5 kb region (chr21:39,883,170–39,861,720) in intron 3 (Figure 1(a)) and enrichment in a second 5 kb region at the end of intron 3 (chr21:39,831,825–39,826,714). Our data from increased sample numbers confirm the results reported by Weier et al. [29]. No correlation was observed between breakpoint positions and age at diagnosis or patient Gleason scores (Figures 1(b) and 1(c)).

### 3.2. Characterization of TMPRSS2-ERG Fusion Sites

**Figure 2:**
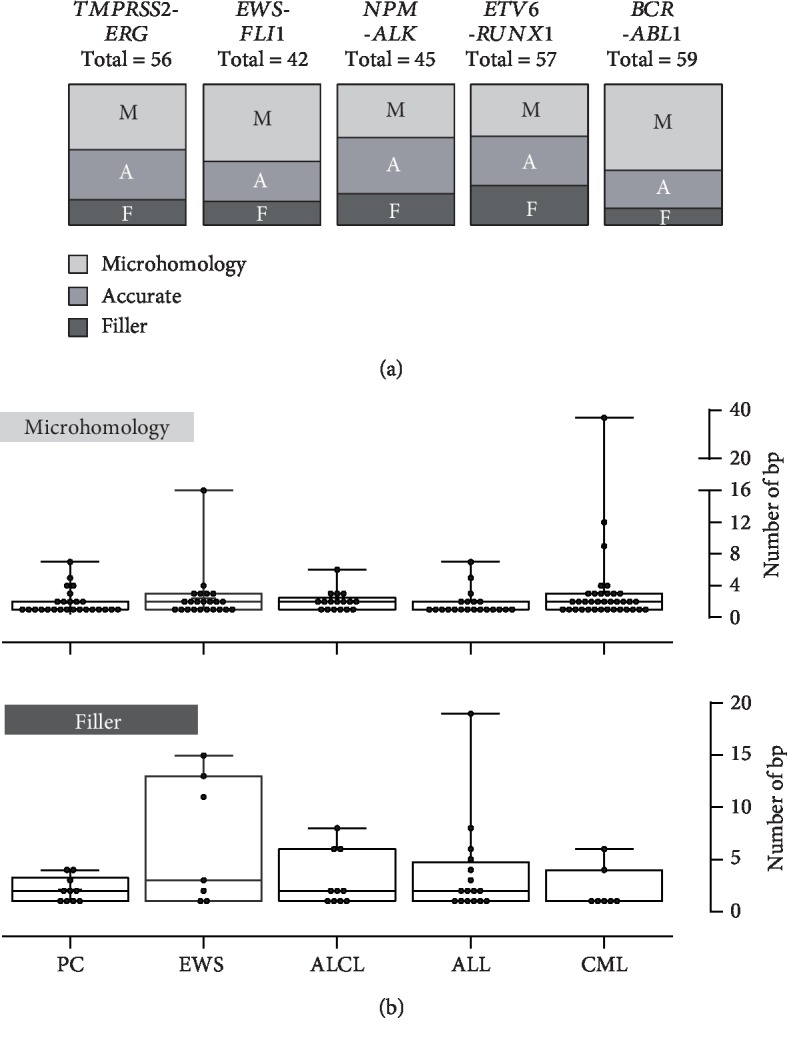
(a) Proportion of accurate transition (A), microhomology (M), and filler (F) events at the fusion sites of chromosomal rearrangements in epithelial prostate cancer (PC) compared with Ewing sarcoma (EWS), anaplastic large-cell lymphoma (ALCL), acute lymphoid leukemia (ALL), and chronic myeloid leukemia (CML). (b) Boxplots representing the median and range of nucleotide numbers involved in microhomology and filler events at the individual fusion sites.

Detailed characterization of the breakpoint fusion sites revealed accurate transitions (36%), small microhomologies (46%), or small fillers (18%) (Figure 2), consistent with the inaccurate nonhomologous end joining repair mechanism that generates chromosomal rearrangements in prostate cancer [29]. Interestingly, the microstructure of genomic fusion sites from epithelial prostate cancer cells was not significantly different from those derived from mesenchymal tumor cells from patients with chronic myeloid leukemia, acute lymphoid leukemia (ALL), anaplastic large-cell lymphoma, and EWS [26, 27, 33, 34] (Figure 2).

### 3.3. Colocalization of TMPRSS2 and ERG Breakpoints within Repeat Elements or DNA-Destabilizing Sequence Motifs

**Figure 3:**
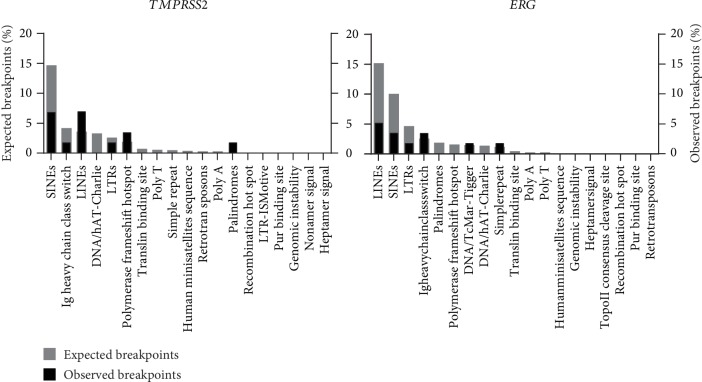
Colocalization of genomic breakpoints with repeat regions and DNA sequence motifs that could support the initiation of chromosomal translocation. Gray columns represent the numbers of expected breakpoints. Black columns represent the numbers of observed breakpoints within the corresponding DNA motif.

We further examined the localization of genomic *TMPRSS2-ERG* fusion sequences with regard to repeat elements and other genomic DNA sequence motifs associated with chromosomal rearrangements (Supplemental [Supplementary-material supplementary-material-1]). No correlation was identified between any specific DNA motif and the localization of genomic fusion breakpoints, including those within the *ERG* breakpoint cluster region (Supplemental [Supplementary-material supplementary-material-1]). Comparison of the expected numbers of breakpoints within a particular DNA sequence motif or repeat element (calculated as the number of bp comprising the DNA motifs within the whole breakpoint cluster region) and the observed numbers of breakpoints within these sequence motifs revealed no significant discrepancy (Figure 3).

Repeat elements at the fusion site impede the design of specific primers for sensitive quantification of tumor-specific circulating DNA copy number. Therefore, we evaluated the distribution of repeat elements within the *TMPRSS2* and *ERG* breakpoint cluster regions to assess the potential limitations of personalized therapy monitoring attributable to sequence composition. The proportion of repeat regions within the breakpoint cluster regions was 25% for *TMPRSS2* and 34% for *ERG*, which is comparable to other mesenchymal tumors for which DNA-level genomic fusion sequences have been successfully established as patient-specific biomarkers (Supplemental [Supplementary-material supplementary-material-1]).

## 4. Discussion

Today, PSA is the most widely used noninvasive tumor marker for evaluation of prostate cancer; however, PSA is not exclusively expressed in malignant tissue. Inflammation, benign prostate hyperplasia, and trauma can also result in increased PSA levels and lead to false positive results [35]. In Germany, approximately 70,000 men are diagnosed with prostate cancer annually, and statistical models predict that prostate cancer will be the most common malignancy by 2030, affecting around 120,000 men per year [36], illustrating the need to improve therapy assessment for this patient cohort. In the present study, we investigated whether genomic *TMPRSS2-ERG* fusion sequences fulfill the molecular criteria for use as patient individual noninvasive tumor markers.

Quantifications of CTCs and cell-free circulating tumor nucleotides in blood or urine samples have been proposed as new molecular strategies for noninvasive tumor monitoring in prostate cancer. The detection of tumor-specific genetic variations enables the establishment of highly specific biomarkers. CTCs are mainly detectable in blood samples from patients with advanced prostate cancer, which carry the complete mutation spectrum from primary tumors and metastases, and therefore represent appropriate biomarkers for advanced-stage disease [15, 16, 37–39]. Cell-free DNA may be more suitable for assessment of therapy effects in patients with early-stage disease [21].

CtDNA has been presented as a valuable biomarker in several other epithelial tumors, including colorectal, breast, lung cancer, and melanoma [40–43], where recurrent point mutations in tumor-suppressor genes or oncogenes were used as molecular markers. Relatively few recurrent point mutations are identified in primary prostate tumors [44]; however, approximately 50% of prostate tumors carry a *TMPRSS2-ERG* translocation [9], which could be considered a highly tumor-specific molecular biomarker for ctDNA quantification in blood or urine samples. The short half-life of ctDNA in blood (less than 2 h [41]) is comparable with that of serum-free PSA [45] and enables real-time therapy assessment.

To use genomic fusion sequences as molecular biomarkers, individual quantification assays are required for each patient because the genomic breakpoints are specific to every individual. Here, we applied nested long-range multiplex PCR to identify patient-specific genomic *TMPRSS2-ERG* fusion sequences. In principle, genomic fusion sequences can also be identified using next-generation sequencing techniques that allow parallel sequencing of several patients in an automated pipeline. Based on the organization of genomic breakpoints in *ERG*, which were preferentially detected within a subcluster region of approximately 25 kb in intron 3, our results provide the basis for the establishment of targeted enrichment assays, including genomic fusion sequences.

In the present study, we analyzed the molecular composition and distribution of genomic *TMPRSS2-ERG* fusion sites from 24 newly sequenced cases together with 34 previously reported patients with prostate cancer to evaluate the suitability of this genomic fusion sequence as a noninvasive tumor marker for patients with prostate cancer. We observed high similarity of the microstructure of genomic fusion sites of epithelial prostate cancer cells compared to mesenchymal tumor cells.

Genomic fusion sequences are considered highly specific tumor markers in leukemic diseases and EWS patients; however, several fusion genes (e.g., *CIC-DUX* in Ewing-like sarcoma and *BCR-ABL1* in Ph+ALL patients) have unfavorable genetic structures for the establishment of high-sensitivity quantification assays. Numerous repeated segments in the *DUX* gene have made the design of tumor-specific primers for therapy assessment in patients with Ewing-like sarcoma impossible. In Ph+ALL patients, the very large breakpoint cluster regions in the fusion genes (55.7 kb in *BCR* and 141 kb in *ABL1*), in combination with the high proportion of repeat elements (50%), complicate the establishment of high-sensitivity quantification assays for therapy assessment. Hence, the localization of genomic breakpoints at repeat elements is crucial for successful application of fusion genes as reliable noninvasive biomarkers, which is especially interesting in the context of ctDNA quantification in patients with solid cancers. Due to the high levels of fragmentation of cell-free DNA in blood plasma or urine samples, to establish highly sensitive assays for therapy assessment primers and probes needs to be positioned close to the fusion site. To ensure that an assay is highly tumor-specific, there should be as few repeat elements as possible associated with the genomic breakpoints.

The present data demonstrate that the proportion of repeat elements in the epithelial tumor, prostate cancer, is comparable to that observed in mesenchymal tumors, where genomic fusion sequences are established as biomarkers (Supplemental [Supplementary-material supplementary-material-1]). Hence, the *TMPRSS2-ERG* fusion gene in prostate cancer could be considered for use as a noninvasive tumor marker for therapy assessment, risk stratification, and relapse detection to improve personalized therapy strategies.

## 5. Conclusions

Large, repeat-rich intronic regions impede the sequencing of genomic *TMPRSS2-ERG* fusion sites. MLR-PCR and next-generation sequencing technologies enable a routine identification of patients' individual fusion sequences. Hence, *TMPRSS2-ERG* fusion sequences are available for the establishment of quantification assays for therapy assessment. The observed comparable proportion of genomic repeat regions within the *TMPRSS2* and *ERG* breakpoint cluster region to other mesenchymal tumors is an important prerequisite for the design of tumor-specific primers and probes for a highly sensitive therapy monitoring in prostate cancer patients.

## Figures and Tables

**Table 1:** Patient characteristics and genomic breakpoint positions.

Patient ID	Age at diagnosis (y)	Gleason score	Break position (GRCh37/hg19)		Filler (bp)	Microhomology (bp)
			*TMPRSS2*	*ERG*		
UPN01	56	6	chr21:42,869,431	chr21:39,882,942	0	1
UPN02	59	7	chr21:42,873,985	chr21:39,853,572	0	1
UPN03	59	7	chr21:42,869,696	chr21:39,829,922 (inversion 43 bp)	0	5
UPN04	60	5	chr21:42,873,983	chr21:39,893,342	0	1
UPN05	61	7	chr21:42,871,961	chr21:39,878,090	0	1
UPN06	62	7	chr21:42,871,305	chr21:39,864,401	3	0
UPN07	63	6	chr21:42,868,091	chr21:39,866,577	0	0
UPN08	64	6	chr21:42,872,946	chr21:39,870,469	4	0
UPN09	65	7	chr21:42,868,907	chr21:39,829,216	2	0
UPN10	66	7	chr21:42,874,680	chr21:39,864,869	0	0
UPN11	66	9	chr21:42,872,341	chr21:39,877,041	0	1
UPN12	67	9	chr21:42,876,941	chr21:39,878,531	0	0
UPN13	68	5	chr21:42,869,152	chr21:39,859,803	0	2
UPN14	68	6	chr21:42,874,909	chr21:39,875,818	0	1
UPN15	68	7	chr21:42,870,630	chr21:39,869,687	0	0
UPN16	69	7	chr21:42,873,489	chr21:39,803,658	0	3
UPN17	69	8	chr21:42,865,245	chr21:39,835,822	0	0
UPN18	69	8	chr21:42,868,095	chr21:39,950,289	0	0
UPN19	70	8	chr21:42,868,866	chr21:39,878,045	1	0
UPN20	70	9	chr21:42,870,028	chr21:39,885,074	0	7
UPN21	72	7	chr21:42,868,434	chr21:39,867,273	0	1
UPN22	72	9	chr21:42,872,857	chr21:39,862,072	0	0
UPN23	73	7	chr21:42,867,920	chr21:39,868,183	0	1
UPN24	74	7	chr21:42,869,364	chr21:39,930,902	0	1
VCap cell line	n.a.	n.a.	chr21:42,871,953	chr21:39,876,353 (inversion 81 bp)	4	0

n.a.: not available.

## Data Availability

The data used to support the findings of this study are available from the corresponding author upon request.

## Supplementary Materials

Supplementary Materials Supplemental Figure 1: genomic breakpoint cluster regions for *TMPRSS2* and *ERG*. Black vertical bars above the breakpoint cluster region represent individual genomic breakpoints in patients with prostate cancer identified in the present study. Orange vertical bars represent individual genomic breakpoints in patients with prostate cancer published in the literature. Localization of repeat elements and different DNA sequence motifs within the breakpoint cluster region are indicated below. Supplemental Figure 2: proportion of repeat regions within the breakpoint cluster regions of fusion genes in prostate cancer (PC), Ewing sarcoma (EWS), anaplastic large-cell lymphoma (ALCL), acute lymphoid leukemia (ALL), and chronic myeloid leukemia (CML). Supplemental Table 1: sequences of primers used in nested multiplex PCR assay. Supplemental Table 2.
