# Supplementary material for: Molecular Composition of Genomic TMPRSS2-ERG Rearrangements in Prostate Cancer
Source: Dis Markers. 2019 Dec 12;2019:5085373. doi: 10.1155/2019/5085373 (PMC6930771; doi:10.1155/2019/5085373)
Supplement: Supplementary Materials — Supplemental Figure 1: genomic breakpoint cluster regions for TMPRSS2 and ERG. Black vertical bars above the breakpoint cluster region represent individual genomic breakpoints in patients with prostate cancer identified in the present study. Orange vertical bars represent individual genomic breakpoints in patients with prostate cancer published in the literature. Localization of repeat elements and different DNA sequence motifs within the breakpoint cluster region are indicated below. Supplemental Figure 2: proportion of repeat regions within the breakpoint cluster regions of fusion genes in prostate cancer (PC), Ewing sarcoma (EWS), anaplastic large-cell lymphoma (ALCL), acute lymphoid leukemia (ALL), and chronic myeloid leukemia (CML). Supplemental Table 1: sequences of primers used in nested multiplex PCR assay. Supplemental Table 2. [file 5085373.f1.pdf]

Supplemental Figure 1

Genomic breakpoint cluster regions for *TMPRSS2* and *ERG*. Black vertical bars above the breakpoint cluster region represent individual genomic breakpoints in patients with prostate cancer identified in the present study. Orange vertical bars represent individual genomic breakpoints in patients with prostate cancer published in the literature. Localization of repeat elements and different DNA sequence motifs within the breakpoint cluster region are indicated below.

Supplemental Figure 2

Proportion of repeat regions within the breakpoint cluster regions of fusion genes in prostate cancer (PC), Ewing sarcoma (EWS), anaplastic large-cell lymphoma (ALCL), acute lymphoid leukemia (ALL), and chronic myeloid leukemia (CML).

Supplemental Figure 1:

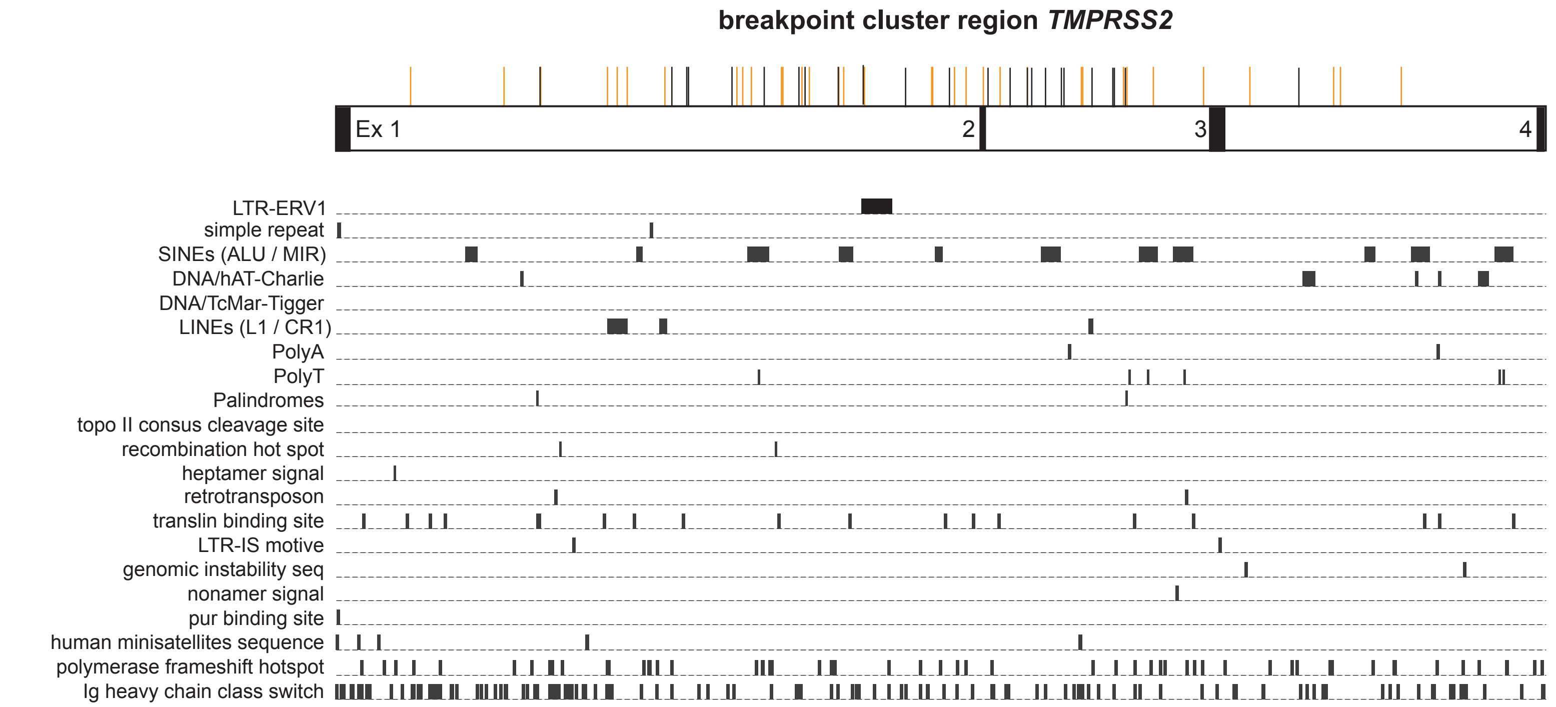

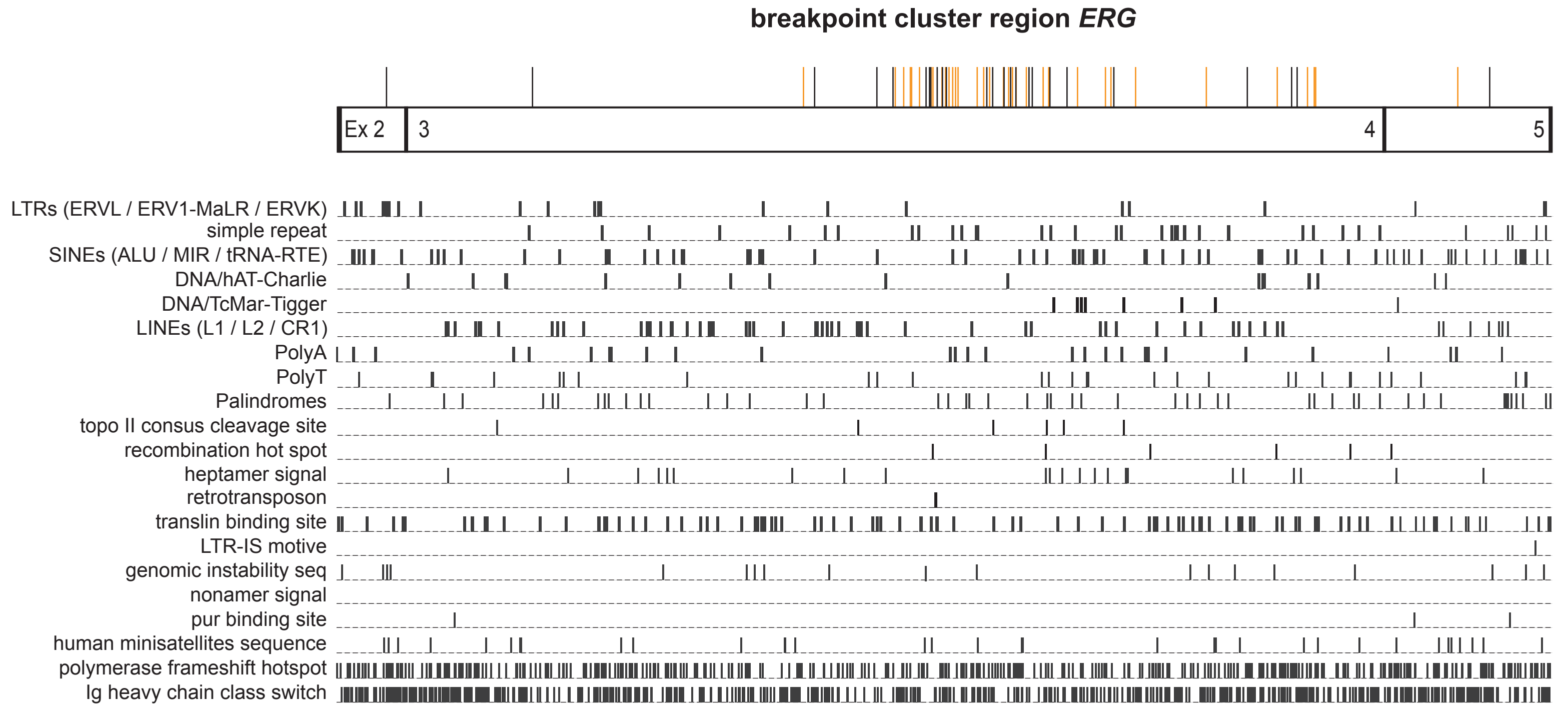

Supplemental Figure 2

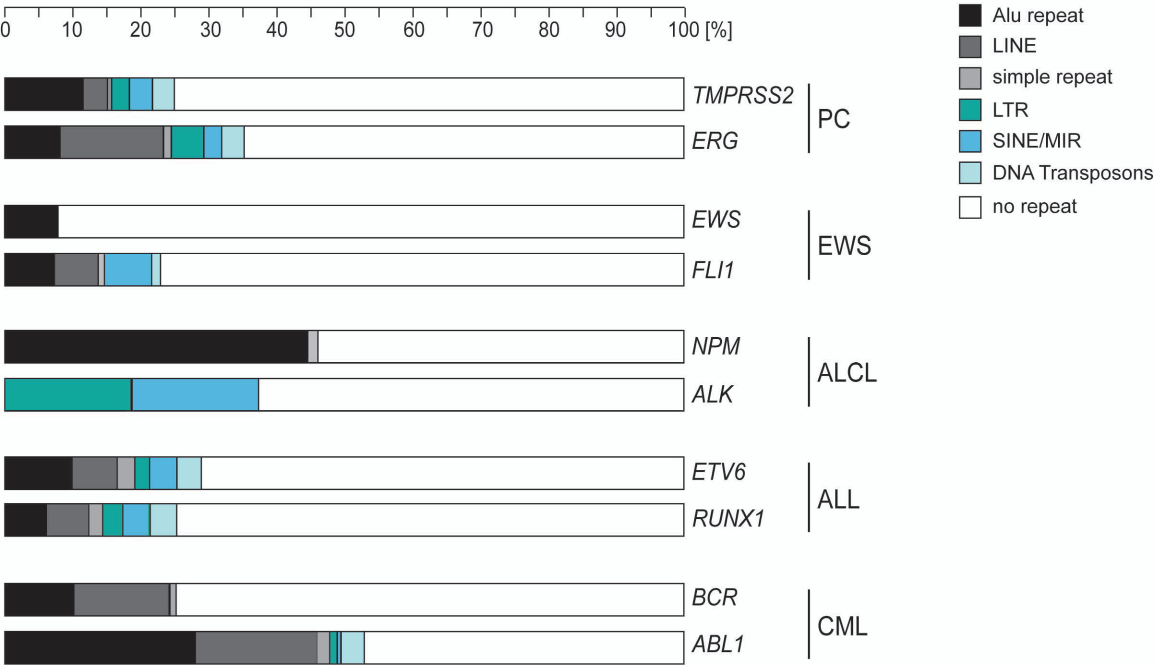

Supplemental Table 1:

Sequences of primers used in nested multiplex PCR assay

Gene		Primer	Sequence 5´-3´	Set
TMPRSS2	external	TMPRSS2_Ex_fw1	gagtaggcgcgagctaagcaggag	Set A
	internal	TMPRSS2_In_fw1	gggctggggaggggaacctg	
	external	TMPRSS2_Ex_fw3	agtgaagtttgagagggcaggaaactcg	
	internal	TMPRSS2_In_fw3	actcgaagacaggcttttcatcctcctg	
	external	TMPRSS2_Ex_fw5	tgcatgtttgttgtaacctgttgggtgag	
	internal	TMPRSS2_In_fw5	ttcatggcaggctgttggggttttatag	
	external	TMPRSS2_Ex_fw7	agggtcctgacgcaggcttcc	
	internal	TMPRSS2_In_fw7	tcgtctgcacgcagcccaaatc	
	external	TMPRSS2_Ex_fw9	tgagcttatcgccctggcagacctc	
	internal	TMPRSS2_In_fw9	aggtgtggccaggtggggagttc	
	external	TMPRSS2_Ex_fw2	cacaggatgcttagaaggggctttagtgac	Set B
	internal	TMPRSS2_In_fw2	gattccttttgatccttccctgggttctc	
	external	TMPRSS2_Ex_fw4	aagggctctttttgatgatttggtcattgg	
	internal	TMPRSS2_In_fw4	attttgaagaaggggcactgagtctgagg	
	external	TMPRSS2_Ex_fw6	actgctcaggaattttcagggacaaacc	
	internal	TMPRSS2_In_fw6	agtggatggcaagttcttagatggctgag	
	external	TMPRSS2_Ex_fw8	gttgatgattgagtaggagcgaacacacag	
	internal	TMPRSS2_In_fw8	atagtcctccaaatgtgcgtgtgaaacaag	
ERG	external	ERG_Ex_rv1	cacctgacctgctggcacgataactc	Set C
	internal	ERG_In_rv1	tgtagctgccgtagttcatcccaacg	
	external	ERG_Ex_rv3	atggaaagaacccgcaatgaaatgg	
	internal	ERG_In_rv3	atggagcctcagcccttgtgagtg	
	external	ERG_Ex_rv5	tgggctcatcttggaagtctgtccatagtc	
	internal	ERG_In_rv5	atctctgtcttagccaggtgtggcgttc	
	external	ERG_Ex_rv7	tcaccccctaaactggttattccacagc	
	internal	ERG_In_rv7	agtcagatgggctagagatacctttattcagagg	
	external	ERG_Ex_rv9	tgagatcttggaggggcaattcttgtc	
	internal	ERG_In_rv9	tgcatcatcaatggagtttaactgtgagagg	
	external	ERG_Ex_rv11	tgcacaatgactgtctaggggcaacag	
	internal	ERG_In_rv11	aggaggaaagcagtcttcattcaaacagg	
	external	ERG_Ex_rv13	agcctatcagggcagggtcctttttcac	
	internal	ERG_In_rv13	accacactagaccagcacagcccatacag	
	external	ERG_Ex_rv15	tccttctgattagagcacattttatggcactg	
	internal	ERG_In_rv15	ctgcataaagagatggctgtgggagtc	
	external	ERG_Ex_rv17	aaattgtatgagtggccttgccctgag	
	internal	ERG_In_rv17	tgaagccagaggttactgaacaactaccc	
	external	ERG_Ex_rv19	tggtaatccagataaattcggagtacaaaggtgag	
	internal	ERG_In_rv19	tgcctggatagcactggctaagaactgc	
	external	ERG_Ex_rv21	tctggagataggaggagatagggcatatgag	
	internal	ERG_In_rv21	tgtggatacaaaaggggtgggccaag	
	external	ERG_Ex_rv23	acttgaccagcagtagctcccacctttc	
	internal	ERG_In_rv23	ttgcagctagaacagcatcagaccaagtg	
	external	ERG_Ex_rv2	tgacaaagagtatgctgtgcagggaatg	Set D
	internal	ERG_In_rv2	tgaagtaggacagtgtgtcaggatgagtgc	
	external	ERG_Ex_rv4	gctttctggtgcgtgaggctagagtg	
	internal	ERG_In_rv4	gaaagggagagggtggctgtggac	
	external	ERG_Ex_rv6	aacctggcttcttcccatttcacctctg	
	internal	ERG_In_rv6	ttccctatgctcagcacatcccttcag	
	external	ERG_Ex_rv8	tgacaggtctgggaaattattcttatggatgg	
	internal	ERG_In_rv8	accaaacaggcacagtctcatgtatgcac	
	external	ERG_Ex_rv10	aggaaaatctggctcttatcccactggag	
	internal	ERG_In_rv10	tggaaatcatcacttgggaggtgttttg	
	external	ERG_Ex_rv12	agtcctgcactgtatttcatgggggaag	
	internal	ERG_In_rv12	cattctcaatcctgctttccagggtcac	
	external	ERG_Ex_rv14	atgtagaggtgcctcagggtcactttgc	
	internal	ERG_In_rv14	accctcctctcccatccctaactttggtc	
	external	ERG_Ex_rv16	aggacaaaagaagctcatgaaagcaaagag	
	internal	ERG_In_rv16	agggtgattgggaaaaatagcctccatac	
	external	ERG_Ex_rv18	ttgccaaatgaacacctgtttcagagtcc	
	internal	ERG_In_rv18	cagagtccctgaaagtcacacatcagaagc	
	external	ERG_Ex_rv20	agcaatctgtgatcacttgagggtgagg	
	internal	ERG_In_rv20	aactggctttccaccaagtaccaccaag	
	external	ERG_Ex_rv22	ttccccaaaatggcaaggagacaaatag	
	internal	ERG_In_rv22	aaggatccgaggacagagaggcaaaattc	
	external	ERG_Ex_rv24	gacacctgccgcccaacctc	
	internal	ERG_In_rv24	actgcagctggattctagggggacctc	

Supplemental Table 2:

Analyzed genomic sequence motif	DNA sequence
DNA polymerase alpha frameshift hotspot	TCCCCC / CTGGCG
DNA polymerase alpha/beta frameshift hotspot	TGGNGT / ACCCCA
DNA polymerase beta frameshift hotspot	ACCCWR
Heptamer recombination signal	CACAGTG
Nonamer recombination signal	ACAAAAACC
Human hypervariable minisatellites sequence 1	GGAGGTGGGCAGGARG
Human hypervariable minisatellites sequence 2	AGAGGTGGGCAGGTGG
Human minisatellites conserved sequence	GCWGGWGG
Human minisatellites core sequence	GGGCAGGARG
Human replication origin consensus	WAWTTDDWWWDHWGWHMAWTT
LTR-IS motif	TGGAAATCCCC
Genomic instability sequence	CCTCCCT
Pur binding site	GGNNGAGGGAGARRRR
Imunoglobuline heavy chain class switch repeats	GAGCT / GGGCT / GGGGT / TGGGG / TGAGC
Recombination hot spot	CCNCCNTNNCCNC
Retrotransposon	TCATACACCACGCAGGGGTAGAGGACT
Translin binding site 1	ATGCAG
Translin binding site 2	GCCCWSSW
Vertebrate topoisomerase II consensus cleavage site	RNYNNCNNGYNGKTNYNY
PolyA (>=10)	AAAAAAAAAA
PolyT (>=10)	TTTTTTTTTT
